# Emergence of Pathogenic Coronaviruses in Cats by Homologous Recombination between Feline and Canine Coronaviruses

**DOI:** 10.1371/journal.pone.0106534

**Published:** 2014-09-02

**Authors:** Yutaka Terada, Nobutaka Matsui, Keita Noguchi, Ryusei Kuwata, Hiroshi Shimoda, Takehisa Soma, Masami Mochizuki, Ken Maeda

**Affiliations:** 1 Laboratory of Veterinary Microbiology, Joint Faculty of Veterinary Medicine, Yamaguchi University, Yamaguchi, Japan; 2 Veterinary Diagnostic Laboratory, Marupi Lifetech Co. Ltd., Osaka, Japan; 3 Laboratory of Emerging Infectious Diseases, Joint Faculty of Veterinary Medicine, Kagoshima University, Kagoshima, Japan; University of Berne, Switzerland

## Abstract

Type II feline coronavirus (FCoV) emerged via double recombination between type I FCoV and type II canine coronavirus (CCoV). In this study, two type I FCoVs, three type II FCoVs and ten type II CCoVs were genetically compared. The results showed that three Japanese type II FCoVs, M91-267, KUK-H/L and Tokyo/cat/130627, also emerged by homologous recombination between type I FCoV and type II CCoV and their parent viruses were genetically different from one another. In addition, the 3′-terminal recombination sites of M91-267, KUK-H/L and Tokyo/cat/130627 were different from one another within the genes encoding membrane and spike proteins, and the 5′-terminal recombination sites were also located at different regions of ORF1. These results indicate that at least three Japanese type II FCoVs emerged independently. Sera from a cat experimentally infected with type I FCoV was unable to neutralize type II CCoV infection, indicating that cats persistently infected with type I FCoV may be superinfected with type II CCoV. Our previous study reported that few Japanese cats have antibody against type II FCoV. All of these observations suggest that type II FCoV emerged inside the cat body and is unable to readily spread among cats, indicating that these recombination events for emergence of pathogenic coronaviruses occur frequently.

## Introduction

Coronaviruses (CoVs) (order *Nidovirales*, family *Coronaviridae*, subfamily *Coronavirinae*) are enveloped and have large single-stranded, positive-sense RNA. Most CoVs cause enteric and/or respiratory diseases in mammals and birds. The 5′ two-thirds of the CoV genome consists of two overlapping open reading frames (ORFs 1a and 1b) that encode a non-structural polyprotein, including RNA-dependent RNA polymerase (RdRp). The other third of the genome consists of ORFs encoding structural proteins, spike (S), membrane (M), envelope (E) and the nucleocapsid (N), and some non-structural proteins (nsp), 3a, 3b, 3c, 7a and 7b [1]. Transcription regulatory sequences (TRS) are located at 5′-distal position in each mRNA and play an important role in the RNA replication of CoV [2], [3].

CoVs frequently undergo mutation and recombination, and there are three reasons for this [4]. First, CoV RdRp has low fidelity. Although CoV encodes nsp14, which possesses 3′→5′ exonuclease activity for proofreading, the mutation rate approaches 2.0×10^−6^ mutations per site per round of replication [5]. Second, there is a unique RNA replication mechanism using the TRS motif that is known as the “copy choice” mechanism, which induces homologous RNA recombination in CoVs [3], [6]. Third, CoV possesses the largest genome (26–32 kb) among RNA viruses. Furthermore, heterologous recombination that *Betacoronavirus* subgroup A has the hemagglutinin esterase gene originated from influenza C virus [7], [8]. These mutation and/or recombination events change viral properties, host range and pathogenicity.

Feline CoV (FCoV) is classified into genus *Alphacoronavirus*, species *Alphacoronavirus 1*, and includes canine CoV (CCoV), transmissible gastroenteritis virus (TGEV) and porcine respiratory CoV (PRCoV). FCoV is distributed worldwide in cats and mainly induces mild intestinal inflammation in kittens [9]. FCoV inducing enteric disease is known as feline enteric coronavirus (FECV). On the other hand, cats infected with FCoV rarely develop the more severe disease feline infectious peritonitis (FIP), which is caused by a mutant virus that is referred to as FIP virus (FIPV). In addition, FCoVs can be divided into two serotypes, types I and II, based on antigenicity [10]–[12]. These serotypes differ primarily in growth characteristics in cell culture and in receptor usage. Type II FCoV is able to use feline aminopeptidase N (fAPN) as its receptor, but type I FCoV cannot [9], [13]. Recently, it was revealed that the S protein was solely responsible for the differences in types I and II FCoV with regard to growth characteristics in cell culture and fAPN usage [14].

CCoV was first isolated in 1971 from dogs with moderate to severe enteritis in Germany [15]. CCoV is widespread in the dog population and is one of the most important canine enteropathogens [16]–[22]. CCoVs were also divided into two genotypes; I and II. Before 2000, it was thought that CCoV had only one genotype, but strain Elmo/02 with a type I FCoV-like S gene was detected in Italy [23]. The Elmo/02 strain possessed a novel ORF3 gene that was absent from other *Alphacoronavirus 1* between the S and ORF3a genes [24]. Finally, this type I FCoV like-CCoV was designated type I CCoV and the reference CCoV was designated type II CCoV. Surprisingly, 36.9%–76.8% of dogs with diarrhea were co-infected with both types I and II CCoV [25]–[27]. Furthermore, type II CCoV was divided into two subtypes, IIa and IIb [28]. In type IIb CCoV, the 5′-terminal region of the S gene was similar to that of TGEV and it was thought that type IIb CCoV emerged via recombination between type IIa CCoV and TGEV [28]. Recently, a type IIa CCoV strain CB/05 with high virulence was reported in Europe [29]. CB/05-infected pups showed clinical signs such as lethargy, vomiting, diarrhea and acute lymphopenia, and the viral genome was observed in extraintestinal tissues including brain [29], [30]. Furthermore, immune response induced by enteric CCoV did not protect dogs from infection with CB/05 [31]. However, there is little genetic information on CCoV in Japan.

In this study, to clarify the mechanisms of emergence of type II FCoV, three type II FCoVs isolated in Japan were genetically and antigenetically compared with ten Japanese type II CCoVs and two Japanese type I FCoVs.

## Materials and Methods

All animal procedures were conducted according to the Yamaguchi University Animal Care and Use guidelines and were approved by the Institutional Animal Care and Use Committee of Yamaguchi University. All efforts were made to minimize pain and suffering.

### Cells

*Felis catus* whole fetus-4 cells (fcwf-4 cells; ATCC Number: CRL-2787) [32] were grown in Dulbecco's modified Eagle's medium (DMEM; Life Technologies, Carlsbad, CA) containing 10% fetal calf serum (FCS), 100 U/ml penicillin and 100 µg/ml streptomycin (Life Technologies). Cells were maintained in a humidified 5% CO_2_ incubator at 37°C.

### Viruses

Type I FCoV strains C3663 and Yayoi, type II FCoV strains M91-267, KUK-H/L and Tokyo/cat/130627 and type II CCoV strains fc1, fc4, fc7, fc9, fc76, fc100, fc94-039, fc97-022, fc00-089 and fc00-016 were analyzed in this study (Table 1). Type I and II FCoVs, excluding Tokyo/cat/130627, were characterized by indirect fluorescence assay (IFA) using monoclonal antibodies (MAbs) that were kindly provided by Dr. Hohdatsu [11], [33]. Yayoi strain was isolated from a cat with a non-effusive form of FIP in Tokyo by serial passage in suckling mouse brain, and was then adapted to fcwf-4 cells [34]. C3663 strain was isolated from a cat with an effusive form of FIP in Kagoshima in 1994 [35]. The pathogenicity of C3663 and Yayoi in cats was characterized [36]. M91-267 strain was isolated from a cat with an effusive form of FIP in Miyazaki in 1991 [35]. Three SPF cats were experimentally infected with M91-267, and all of these died from FIP (unpublished data). KUK-H strain was isolated from a cat with an effusive form of FIP in Kagoshima in 1987, and KUK-H/L that formed large plaques was plaque-purified from the KUK-H strain [35]. KUK-H/L caused lethal FIP in cats [35]. RNA sequences of Tokyo/cat/130627 were obtained from FIP ascites in a cat in Tokyo in 2013. The FIPV spread quickly in a cattery, and more than twenty cats developed FIP. Type II CCoV strains, fc1, fc4, fc7, fc9, fc76, fc100, fc94-039 and fc97-022, were isolated between 1990 and 1997 in Japan [19], and fc00-016 and fc00-087 were isolated in 2000 in Japan [37].

**Table 1:** Canine and feline coronaviruses analyzed in this study: nucleotide sequence acquisition numbers and serum cross-neutralizing activity.

Virus	Strain	Accession No.				VN titers	
		RdRp	S - poly A	Partial S	N	Type I^a^	Type II^b^
Type II CCoV	fc1	AB781791	AB781790	AB781790	AB781790	<1∶10	1∶400
	fc4	AB907625		AB781807	AB781797	<1∶10	1∶4525
	fc7	AB907626		AB781808	AB781798	<1∶10	1∶1600
	fc9	AB907627		AB781809	AB781799	<1∶10	1∶1600
	fc76	AB907628		AB781810	AB781800	<1∶10	1∶9051
	fc100	AB907629		AB781811	AB781801	<1∶10	1∶1131
	fc97-022	AB907631		AB781812	AB781802	<1∶10	1∶2263
	fc94-039	AB907630		AB781813	AB781803	<1∶10	1∶2263
	fc00-016	AB907632		AB781814	AB781804	<1∶10	1∶1600
	fc00-089	AB907633		AB781815	AB781805	<1∶10	1∶200
Type II FCoV	M91-267	AB781792	AB781788	AB781788	AB781788	<1∶10	1∶25600
	KUK-H/L	AB781793	AB781789	AB781789	AB781789	<1∶10	1∶6400
	Tokyo/cat/130627	AB907634	AB907624	AB907624	AB907624	N.D.	N.D.
Type I FCoV	C3663	AB781794	AB535528^c^	AB535528^c^	AB535528^c^	1∶6400	1∶80
	Yayoi	AB781795		AB695067^c^	AB781806	1∶2000	1∶160

N.D.: Not done.

a Serum was collected from the cat that was inoculated intraorally with type I FCoV C3663 [36].

b Serum was collected from the cat that was inoculated intraperitoneally with type II FCoV M91-267 (unpublished data).

c [36].

### Reverse transcription (RT)-polymerase chain reaction (PCR)

Each virus, excluding Tokyo/cat/130627, was inoculated onto an fcwf-4 cell monolayer and was incubated until cytopathic effects (CPEs) were observed. RNA was then extracted from fcwf-4 cells using an RNeasy Mini kit (Qiagen, Hilden, Germany) and RT reaction was carried out at 30°C for 10 min, 42°C for 30 min, 70°C for 15 min and 5°C for 5 min with random 9-mer oligonucleotide primers or 42°C for 30 min, 70°C for 15 min and 5°C for 5 min with oligo dT-adaptor primer using a TaKaRa RNA LA PCR kit (AMV) Ver.1.1 (TaKaRa, Shiga, Japan).

For amplification of partial S genes of type II CCoVs and type II FCoVs, primers CCVSF (5′-AGCACTTTTCCTATTGATTG-3′) and CCVSR (5′-GTTAGTTTGTCTAATAATACCAACACC-3′) were used [38]. For amplification of the N gene, primers NF (5′-CTAAAGCTGGTGATTACTCAACAG-3′) and NR (5′-TAATAAATACAGCGTGGAGGAAAAC-3′) were used [39]. PCR was carried out at 94°C for 2 min, followed by 40 cycles at 94°C for 30 s, 55°C for 30 s, 72°C for 2 min and final extension at 72°C for 10 min using a TaKaRa RNA LA PCR kit (AMV) Ver.1.1 (TaKaRa). PCR products were analyzed electrophoretically and amplified products were purified using a QIAquick PCR Purification kit (Qiagen) for sequence analysis.

In order to amplify the subgenomic mRNA of CCoV fc1, PCR was performed using 52F (5′-ACTAGCCTTGTGCTAGATTT-3′) as a forward primer and CCVScenR (5′-CCAGTTTTTATAACAGCTG-3′), N-RR2 (5′-GCGCAATAACGTTCACCA-3′) and M13 primer M4 as reverse primers. Primer 52F recognized the TRS conserved among *Alphacoronavirus*es [36]. The reaction was carried out under the same conditions as mentioned above.

For sequence analysis of ORFs M, N, 7a and 7b of M91-267 and KUK-H/L, we carried out TA cloning. RNA was extracted from fcwf-4 cells infected with M91-267 or KUK-H/L using an RNeasy Mini kit (Qiagen). Extracted RNA was reverse-transcribed with oligo dT-Adaptor primer using a TaKaRa RNA LA PCR kit (AMV) Ver.1.1 (TaKaRa) as mentioned above. To amplify the region including ORFs M, N, 7a and 7b, primers 52F, M13 primer M4 were used for PCR with a TaKaRa RNA LA PCR kit (AMV) Ver.1.1 (TaKaRa). PCR products were directly cloned into pGEM-T Easy (Promega, Madison, WI) according to the manufacturer's instructions. Plasmid DNAs were extracted from *E. coli* strain JM109 using a QIAprep Spin Miniprep Kit (Qiagen). Purified plasmid DNAs were applied for sequencing analysis.

Viral RNA of Tokyo/cat/130627 was extracted from FIP ascites in a cat using a QIAamp Viral RNA Mini Kit (Qiagen). For sequence analysis, five fragments of the Tokyo/cat/130627 gene between the 3′- terminus of ORF 1b and poly A were amplified using the following primer pairs: 1bF (5′-TTGATTCAAAGATTTGAGTATTGG-3′)-CCVSR; CCVSF-S2cenFR3 (5′-GTGTCAATTCAGGTACAG-3′); S2cenFF2 (5′-GAGTGCTGATGCACAAGT-3′)-N-RR3 (5′-GCCACCATACAATGTGAC-3′); N-RF4 (5′- AGTTCAGCATTGCTGTGCTC-3′)-N4 (5′-CATCTCAACCTGTGTGTCAT-3′); and N1 (5′-MMAAYAAACACACCTGGAAG-3′)-oligo dT-Adaptor primer. RT-PCR was carried out using a QIAGEN OneStep RT-PCR Kit (Qiagen) according to the manufacturer's instructions. Reactions were carried out at 45°C for 45 min and 95°C for 15 min, followed by 40 cycles at 94°C for 10 s, 55°C for 30 s, 68°C for 3 min, and a final extension at 68°C for 15 min. For amplification of partial RdRp genes, primer IN-6 (5′- GGTTGGGACTATCCTAAGTGTGA -3′) and IN-7 (5′- CCATCATCAGATAGAATCATCAT -3′) were used as described previously [40]. PCR products were analyzed electrophoretically and amplified products were purified using a MinElute PCR Purification Kit (Qiagen) for sequence analysis.

### Nucleotide sequences

Sequencing was performed using a BigDye Terminator v3.1 Cycle Sequencing kit (Life Technologies) according to the manufacturer's instructions. Products were purified by ethanol precipitation and analyzed using an ABI PRISM 310 Genetic Analyzer (Life Technologies). For sequence analysis, primers shown in Table S1 were used and nucleotide sequences were deposited to the DNA database of Japan (DDBJ) under the accession numbers listed in Table 1.

### Homology search and phylogenetic analysis

Homologies among strains were analyzed using GENETYX Ver.8 (GENETYX Corporation, Tokyo, Japan) and phylogenetic trees were constructed by the neighbor-joining method [41] using MEGA5.0 software [42] based on nucleotide pairwise distance. For construction of the phylogenetic tree, we referred to the following sequences; type II FCoV 79-1146 (accession no. DQ010921), 79-1683 (JN634064), DF-2 (JQ408981) and NTU156/P/2007 (GQ152141), type I FCoV C3663 (AB535528), Yayoi (AB695067 for S), UCD1 (AB088222 for S, AB086902 for N), Black (EU186072), NTU2/R/2003 (DQ160294), RM (FJ938051), UCD11a (FJ917519), UCD5 (FJ917522), UCD12 (FJ917521), UCD13 (FJ917523), UCD14 (FJ917524), UU2 (FJ938060), UU16 (FJ938058), UU18 (HQ012368), UU20 (HQ392471), UU21 (HQ012369), UU23 (GU553362), type II CCoV 1-71 (JQ404409), v1 (AY390342 for S, AY390345 for N), K378 (KC175340), NTU336/F/2008 (GQ477367), 5821 (AB017789 for S), TGEV Purdue (DQ811789), and PRCoV ISU-1 (DQ811787). Analysis of the similarity in the 3′-region of the genome, excluding poly A, was carried out using Simplot version 3.5.1 [43].

### Sera from cats

Sera collected from two SPF cats experimentally inoculated with FIPVs were used. One cat was inoculated intra-orally with type I FCoV C3663 (3.9×10^6^ PFU/cat) and showed an effusion form of FIP [36]. Another cat was inoculated intraperitoneally with type II FCoV M91-267 (1.0×10^6^ PFU/cat) and also showed an effusive form of FIP (unpublished data). When clinical symptoms were severe, cats were euthanized under anesthesia. These sera were obtained in our previous experiments carried out under approval by the ethics committee for animal experiments, Faculty of Agriculture, Yamaguchi University.

### Virus-neutralization test

Virus-neutralization (VN) test was performed by 75% plaque-reduction neutralization test (PRNT_75_) using cat sera inactivated at 56°C for 30 min [12], [36]. Equal volumes of two-fold serially diluted sera and 2.0×10^3^ PFU/ml virus were mixed and incubated at 37°C for 1 h. Then, 50 µl of this mixture was inoculated onto an fcwf-4 cell monolayer in 24-well plates (Sumitomo Bakelite, Tokyo, Japan). After adsorption at 37°C for 1 h, inoculum was removed and 0.8% agarose (Seaplaque GTG Agarose; Lonza, Switzerland) in DMEM containing 10% FCS was overlaid. Infected cells were incubated at 37°C until CPE was observed, followed by fixing with phosphate-buffered formalin and staining with crystal violet.

## Results

### Comparison of 3′-region among type II CCoVs, and type I and II FCoVs

**Figure 1:**
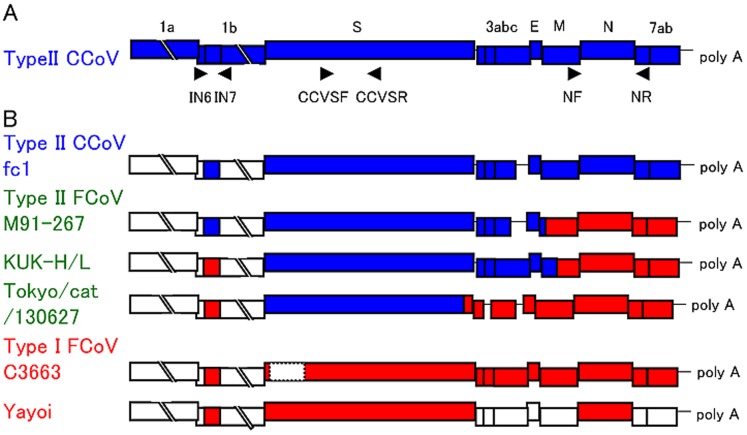
Schema of feline and canine coronaviruses. (**A**) Schema of type II CCoV. Each ORF is indicated by squares. Arrowheads indicate location of primers for amplification of partial RdRp, partial S and full N genes. (**B**) Schema of type II CCoV fc1, type II FCoV M91-267, KUK-H/L and Tokyo/cat/130627, and type I FCoV C3663 and Yayoi. Blue boxes indicate ORFs originating from type II CCoV. Red boxes indicate ORFs originating from type I FCoV.

Nucleotide sequences of the 3′-region of the genomes, excluding the poly A, of type II CCoV fc1 (8,959b) and type II FCoVs, M91-267 (8,889b), KUK-H/L (8,930b) and Tokyo/cat/130627 (8,831b), were determined (DDBJ Accession No. AB781790 for fc1, AB781788 for M91-267, AB781789 for KUK-H/L and AB907624 for Tokyo/cat/130627) (Table1). Because of a mutation in the start codon (ATG→ACG), Tokyo/cat/130627 lacked ORF3b. In addition, type II FCoVs, M91-267 and Tokyo/cat/130627 possessed a truncated ORF 3c (Fig. 1). When compared with KUK-H/L, M91-267 had a 35-nucleotide deletion in the ORF 3c gene, resulting in a truncated ORF 3c. In comparison with C3663, Tokyo/cat/130627 showed a 25-nucleotide deletion in the ORF 3c gene, resulting in a truncated ORF 3c gene. Deduced amino acid sequences for ORFs S, 3a, 3b, 3c, E, M, N, 7a and 7b in type II FCoVs were compared with those of type I FCoV C3663 and type II CCoV fc1 (Table S2, S3). Both M91-267 and KUK-H/L showed low identities with type I FCoV C3663 in ORFs S, 3a, 3b, 3c and E and high identities in ORFs N, 7a and 7b (Table S2). In contrast, the two strains showed high identities with type II CCoV fc1 in ORFs S, 3a, 3b, 3c and E and low identities in ORFs N, 7a and 7b (Table S3). In ORF M, the identities among type I FCoV, type II FCoV and CCoV were neither high nor low (Table S2, S3). Interestingly, comparison between Tokyo/cat/130627 and type I FCoV showed low identities in ORF S and high identities in ORFs 3a, 3c, E, M, N, 7a and 7b, while comparison with type II CCoV fc1 showed high identity only in ORF S and low identities in ORF 3a, 3c, E, M, N, 7a and 7b (Table S2, S3).

### Comparison of partial RdRp genes among type II CCoVs and type I and II FCoVs

**Figure 2:**
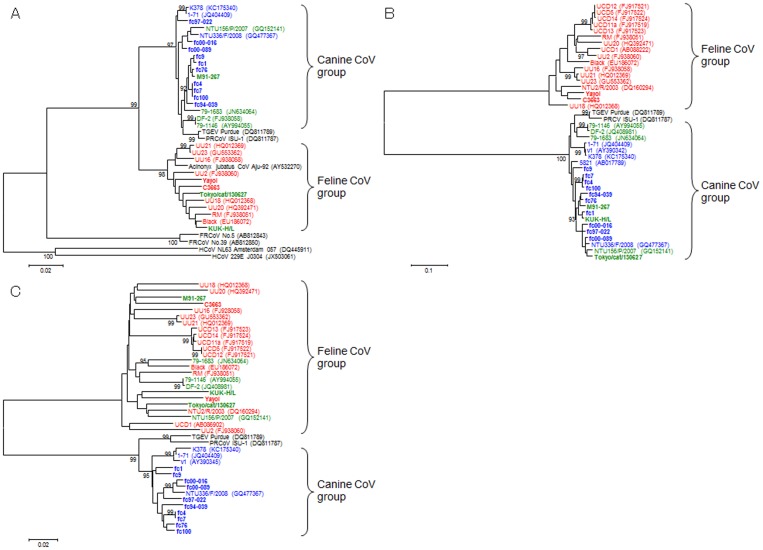
Phylogenetic trees using partial RdRp(A), partial S (B) and N (C) genes. Type I FCoVs, type II FCoVs and type II CCoVs are shown in red, green and blue, respectively. Swine CoV (TGEV and PRCoV), ferret CoV (FRCoV) and human CoV (HCoV) are shown in black. GenBank accession numbers are shown in parentheses.

Nucleotide sequences of partial RdRp gene in ORF1b (394b) of 15 Japanese CoVs were determined and deduced amino acid sequences were compared (Tables S2, S3, S4 and Fig. 2A). In comparison with type I FCoVs, C3663, KUK-H/L and Tokyo/cat/130627 showed higher identity in RdRp than M91-267 (Table S2). On the other hand, the sequence of RdRp of M91-267 was more similar to that of type II CCoV fc1 than type I FCoV C3663 (Table S3). All CCoV strains possessed high homology with fc1 strain and M91-267, but showed low homology with KUK-H/L and Tokyo/cat/130627 (Table S4).

Phylogenetic analysis using partial RdRp genes showed that Japanese type II strains could be divided into two different groups; feline CoV and canine CoV (Fig. 2A). KUK-H/L and Tokyo/cat/130627 belonged to feline CoV group and M91-267 belonged to canine CoV group. The other foreign type II FCoVs belonged to the type II CCoV group.

### Comparison of partial S genes among type II CCoVs and type I and II FCoVs

Nucleotide sequences of partial S genes (692b) of 15 Japanese CoVs were determined and deduced amino acid sequences were compared (Table S5 and Fig. 2B). In comparison with type I FCoV C3663, all type II FCoVs showed low identity. All CCoV strains possessed high homology with fc1 strain and type II FCoVs, but showed low homology with type I FCoV C3663 (Table S5).

Phylogenetic analysis using partial S genes showed that all type II FCoVs were more similar to type II CCoV than type I FCoV (Fig.2B). Furthermore, Japanese type II FCoVs were more similar to Japanese type II CCoV than type II FCoVs and type II CCoVs from other countries. In addition, Japanese FCoVs belonged to different subgroups; KUK-H/L belongs to a cluster with fc1. M91-267 belongs to the other cluster with fc76 and fc94-039. Tokyo/cat/130627 belongs to the cluster with Taiwanese strain NTU156/P/2007 (Fig. 2B).

### Comparison of N genes among type II CCoVs and type I and II FCoVs

Nucleotide sequences of N genes (1149b) of 15 Japanese CoVs were determined and deduced amino acid sequences were compared (Tables S2, S3, S6 and Fig. 2C). In comparison with type I FCoV C3663, all type II FCoVs showed higher identity than type II CCoV fc1 (Table S2). All CCoV strains possessed high homology with fc1 strain, but showed low homology with types I and II FCoV (Table S6).

Phylogenetic analysis using N genes showed that FCoV strains and type II CCoV strains were genetically divided into different groups. In the feline CoV group, Japanese type II FCoVs M91-267, KUK-H/L and Tokyo/cat/130627 belonged to different clusters (Fig. 2C). KUK-H/L was similar to Yayoi, M91-267 was similar to C3663, and Tokyo/cat/130627 was similar to Taiwanese strain NTU2/R/2003 (Fig. 2C). Japanese CCoVs formed one cluster with Taiwanese strain NTU336/F/2008.

### Recombination sites of type II FCoVs

**Figure 3:**
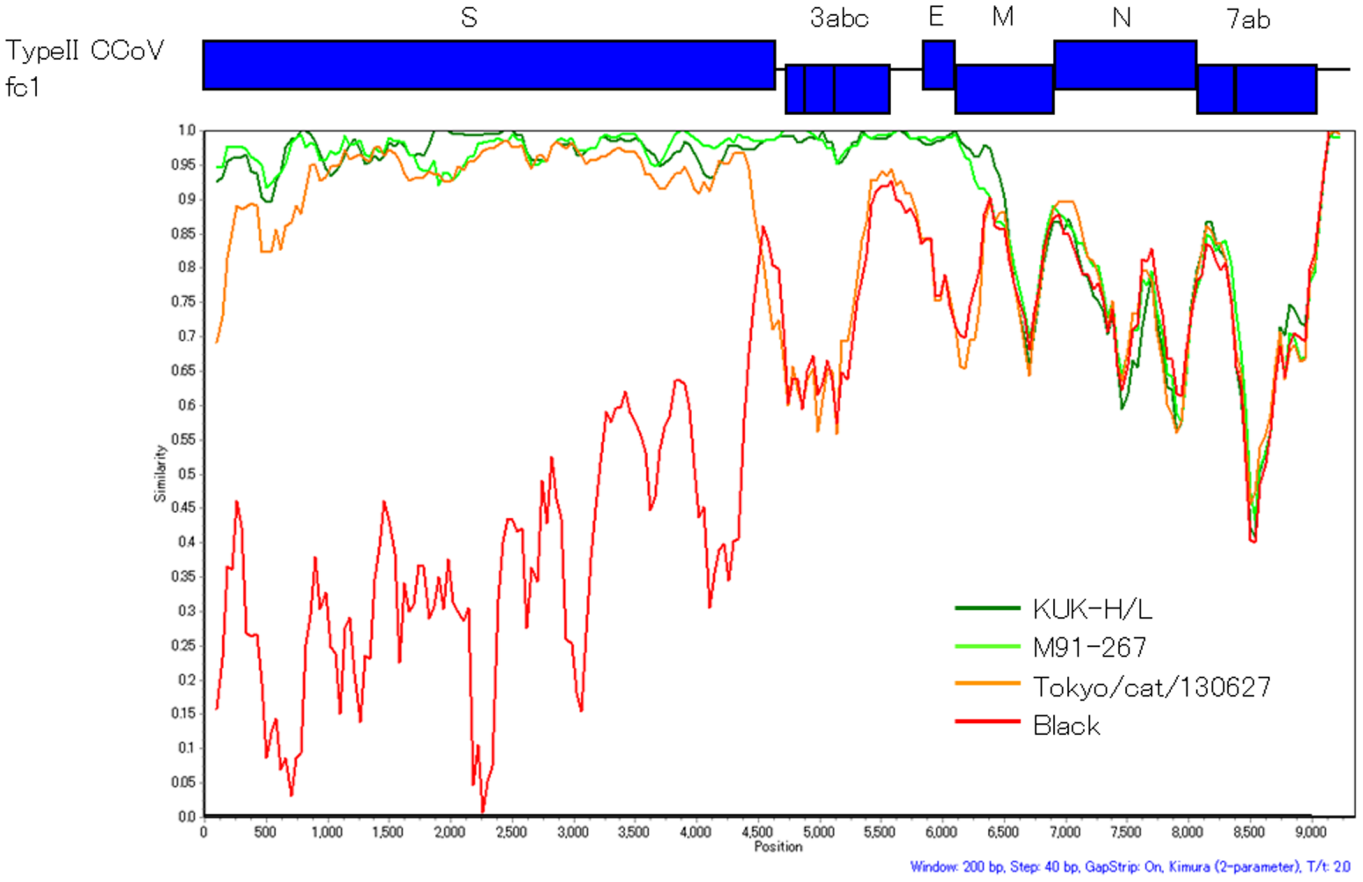
Simplot analysis of canine and feline coronaviruses. Similarity between nucleotide sequences of 3′-region of genome of type II CCoV fc1, type I FCoV Black, and type II FCoVs KUK-H/L, M91-267 and Tokyo/cat/130627. Horizontal axis refers to nucleotide position of fc1. Upper region of the plot map shows ORF structure in type II CCoV fc1 and corresponds to nucleotide positions in the plot map. A similarity of 1.0 indicates 100% identity with the nucleotide sequence. Parameters for calculation were as follows: window size, 200 bp; and step size, 40 bp.

Simplot analysis showed that the similarity of Tokyo/cat/130627 to CCoV fc1 changed at the 3′-terminal region of the S gene, and those of M91-267 and KUK-H/L changed within the M gene (Fig. 3).

**Figure 4:**
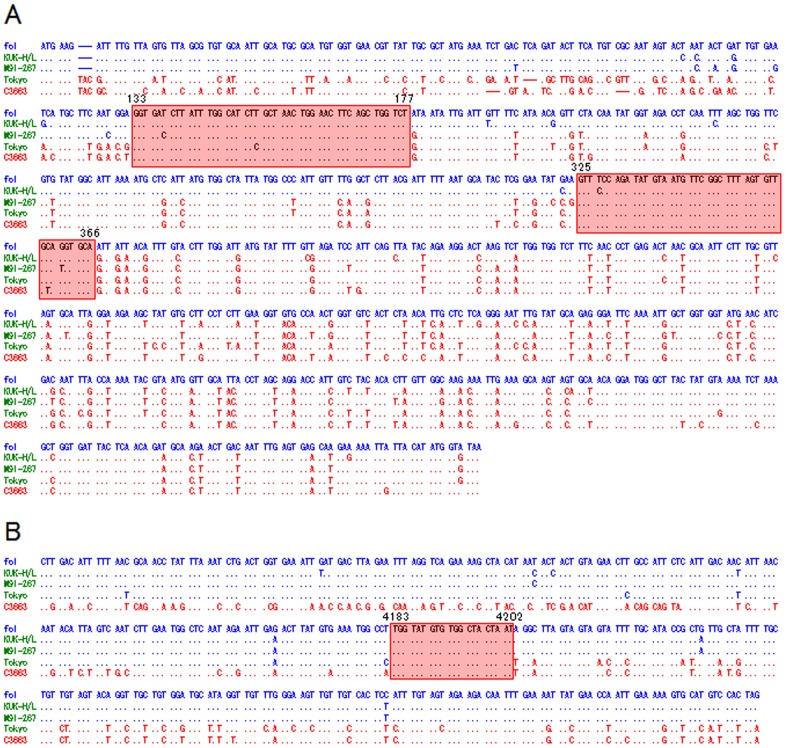
Alignment of M and 3′-terminal of S genes in canine and feline coronaviruses. (**A**) Alignment of M genes of CCoV and FCoV strains. Two regions in squares are conserved regions among type II CCoV fc1, type II FCoVs M91-267, KUK-H/L and Tokyo/cat/130627 and type I FCoV C3663. (**B**) Alignment of 3′-terminal of S genes of CCoV and FCoV strains. Square indicates conserved region. Nucleotide sequences originating from type II CCoV and type I FCoV are shown in blue and red, respectively. Dots indicate the same sequences with type II CCoV fc1.

The M genes were compared among types I and II FCoV and type II CCoV (Fig. 4A). The alignment showed that the 5′-terminal region of the M genes of M91-267 and KUK-H/L was similar to that of CCoV fc1, but the 3′-terminal region was similar to type I FCoV C3663 (Fig. 4A). The M gene of Tokyo/cat/130627 was similar to type I FCoV C3663. Furthermore, the nucleotide sequences indicated that the recombination sites of these two viruses, M91-267 and KUK-H/L, were different. Among these CoVs, two conserved regions were located at 133–177 and 325–366 in the M gene. KUK-H/L was similar to type II CCoV upstream of the first conserved region (region 133–177), but was similar to type I FCoV downstream of the region. On the other hand, M91-267 was similar to type II CCoV upstream of the second conserved region (region 325–366), and was similar to type I FCoV downstream of the region.

The alignment data using type I FCoV C3663, type II FCoV M91-267, KUK-H/L and Tokyo/cat/130627, and type II CCoV fc1 showed that the recombination site of Tokyo/cat/130627 was in the 3′-terminal of the S gene. Among these FCoVs and CCoVs, region 4183–4202 of the S gene was completely conserved (Fig. 4B). Upstream of the conserved region, Tokyo/cat/130627 was more similar to type II CCoV fc1 than type I FCoV C3663, and downstream of the conserved region, Tokyo/cat/130627 was more similar to type I FCoV C3663 (Fig. 4B).

### Cross-neutralization activity to CCoV by sera collected from cats infected with FCoV

In order to examine whether cats with VN antibody against type I FCoV can be infected with type II CCoV, cross-neutralizing activity of sera from cats experimentally infected with FCoVs was examined (Table 1). Cat serum against type I FCoV C3663 was able to neutralize infection of type I FCoV strains C3663 and Yayoi (1∶6400 and 1∶2000, respectively), but not those of type II CCoV and type II FCoV (less than 1∶10) (Table1). On the other hand, cat serum against type II FCoV M91-267 was able to neutralize infection of type II FCoV (1∶6400–1∶25600), CCoV (1∶200–1∶9051) and type I FCoV (1∶80–1∶160) (Table1).

## Discussion

Type II FCoV emerged as a result of recombination events between type I FCoV and type II CCoV [44], [45]. Recently, one additional full genome sequence of type II FCoV NTU156/P/2007 was determined, and this facilitated understanding of the mechanisms responsible for emergence of type II FCoV [46]. The prevalence of type II FCoV in the cat population is lower than that of type I, but the reasons for this remain uncertain [12], [47]–[51]. In this study, numerous FCoV and CCoV isolates from Japan were genetically characterized, and the emergence of type II was discussed.

Our phylogenetic and sequence analysis clearly indicated that type II FCoVs emerged by different recombination events between type I FCoV and type II CCoV. In addition, other type II FCoVs isolated from the USA (79–1683 and 79–1146) and Chinese Taipei (NTU156/P/2007) also showed different origins [45], [46]. These results indicate that type II FCoV independently emerged in different cats and did not spread very easily. Our previous study also showed that many cats possess VN antibody to type I FCoV, but few cats in Japan possess VN antibody against type II FCoV [12], supporting the notion that type II FCoV does not readily spread among the cat population. The reasons why type II FCoV is unable to spread among the cat population are unclear.

Two of three stains of Japanese type II FCoV, M91-267 and Tokyo/cat/130627, possessed the truncated ORF 3c gene (Fig. 1). An intact 3c gene is apparently essential for efficient replication of FCoV in the intestinal tract, resulting in the secretion of FCoV from feces and transmission of FCoV among cat population [55], [56]. On the other hand, many FIPV possessed truncated 3c gene and cats with FIP did not excrete virus in feces [57]–[59]. Furthermore, one outbreak of type II FIPV with intact ORF 3c gene occurred in Taiwan. In early stages of the outbreak, the type II FIPV possessed intact 3c gene, but lost it in later stages [60]. Therefore, these type II FCoVs with truncated 3c gene, M91-267 and Tokyo/cat/130627, might not spread well among cats.

It is interesting that all FCoVs, both types I and II, possessed the 5′- and 3′-termini of the FCoV genome, but not CCoV. These regions may be essential for growth of FCoV in cats and double recombination may be required to maintain both the 5′- and 3′-termini of FCoV. Type II FCoVs possessed two types of RdRp element derived from type I FCoV or type II CCoV (Fig. 2A), suggesting both types of RdRp were able to function during replication and transcription in cat body. Furthermore, the region upstream of RdRp might be essential for FCoV infection in cats. In addition, it has been reported that N protein is important for viral particle production [52], and the N gene is conserved among FCoVs. Therefore, the N protein of FCoV, but not CCoV, may be essential for replication of FCoV in cats. Interestingly, simplot analysis showed four other candidate recombination sites, one in the 3c gene, two in the N gene and one in the 7a gene, which showed high identity between CCoV and type I FCoV (Fig. 3). If the M or N genes of type I FCoV are not necessary for growth of FCoV in cats, other recombinant type II FCoVs using these possible recombination sites must occur. Further analysis of type II FCoV is necessary to clarify the recombination events of CoV in cats.

Four full genome sequences of type II FCoVs (79–1146, 79–1683, DF-2 and NTU156/P/2007) are deposited in GenBank. We also reported one-third of the full genome of three type II FCoV strains (M91-267, KUK-H/L and Tokyo/cat/130627) and one type II CCoV fc1. Six of seven type II FCoV strains emerged by recombination events at the E or M gene. However, the recombination event of Tokyo/cat/130627 occurred at the 3′-terminal of the S gene. The nucleotide sequences indicated that M91-267, KUK-H/L and Tokyo/cat/130627 originated from type I FCoV strains similar to C3663, Yayoi and NTU2/R/2003, respectively, and that the central region, including the S gene, was acquired from type II CCoV strains similar to fc94-039, fc1 and fc00-089, respectively. In addition, the recombination sites were clearly different (Fig.3 and 4A, B). These results indicate that the recombination events between type I FCoV and type II CCoV occurred independently. In addition, original viruses of foreign type II FCoVs, 79-1146, 79-1683 and NTU156/P/2007 differed from those of these three Japanese type II FCoVs, indicating that the recombination events occurred among cat populations all over the world.

Sera from cats experimentally inoculated with type I FCoV C3663 could not neutralize type II CCoV infection (Table 1), thus suggesting that the cat infected with type I FCoV could not prevent type II FCoV infection. On the other hand, the cat infected with type II FCoV could neutralize type I FCoV infection (Table 1). In addition, many sera from type II FCoV-infected cats in the outbreak could cross-neutralize type I FCoV infection, and those from type I FCoV-infected cats in the field could not cross-neutralize type II FCoV infection (our unpublished data). These results suggest that the cross-reactivity to type I FCoV in type II FCoV-infected cats might be induced by viral proteins other than S protein. Further analysis will be required to clarify the cross VN activity in type II FCoV-infected cats.

**Figure 5:**
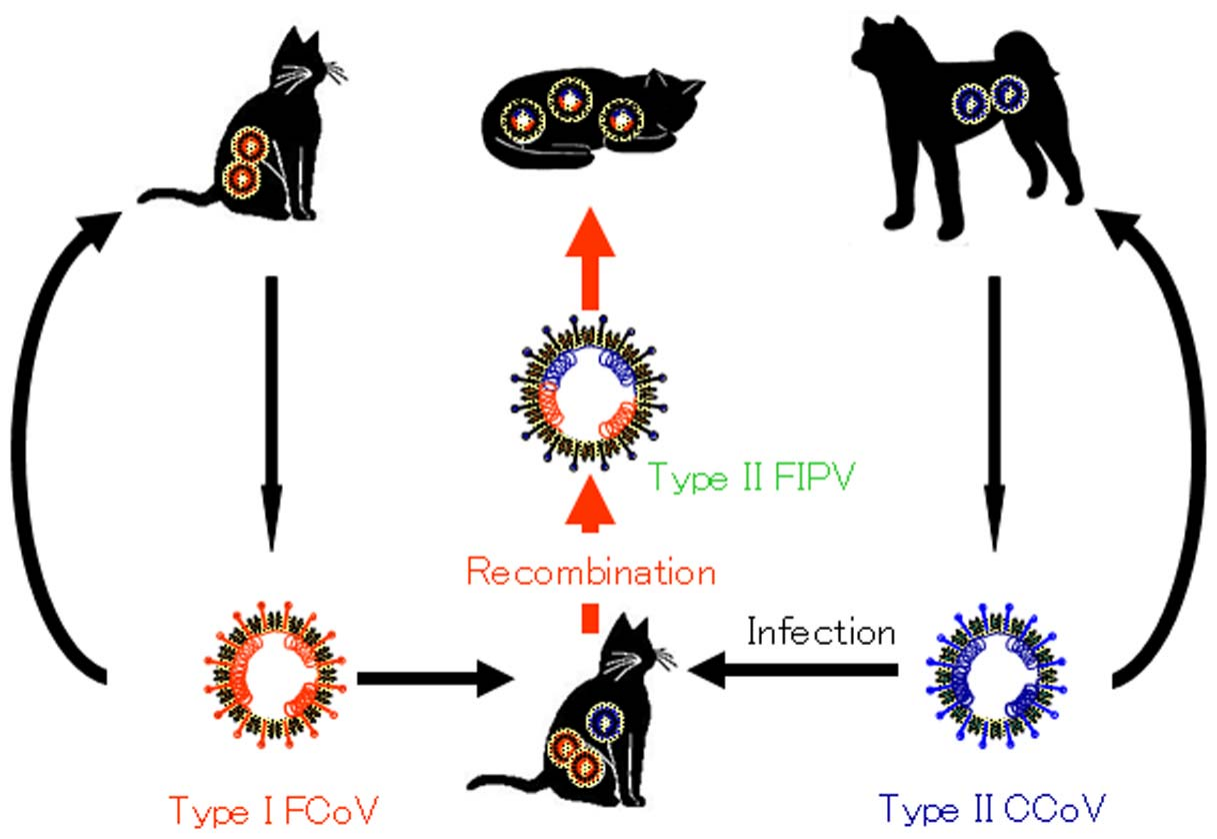
Hypothesis of emergence of type II FCoV. Some cats persistently infected with type I FCoV are superinfected with type II CCoV which is excreted from dogs. Inside the cat body, type II FCoV emerges by homologous recombination and induces severe clinical disease, FIP. Diseased cats do not spread type II FCoV.

Type II CCoV was able to use “feline” aminopeptidase N as a receptor to infect cats [53], [54] and type I FCoV-infected cats did not possess VN antibody against type II CCoV infection (Table 1), indicating that cats infected with type I FCoV could be superinfected with type II CCoV from dogs. Our hypothesis on the mechanism of emergence of type II FCoV is shown in Fig. 5. Cats infected with type I FCoV were unable to produce VN antibody against type II CCoV. Hence, cats had the possibility of superinfection with type II CCoV. The recombination event between type I FCoV and type II CCoV occurred inside the cat body, leading to emergence of type II FCoV.

CoVs, such as SARS-CoV, tend to change their host range by mutation and/or recombination [61]. Homologous recombination is a significant factor for change of host range. Therefore, investigations into homologous recombination of CoVs may help to clarify the mechanisms responsible for changes in host range.

## Supporting Information

Table S1 Primers used in this study.
(DOCX)

Table S2 Comparison of ORF identities between C3663 and other coronaviruses.
(DOCX)

Table S3 Comparison of ORF identities between fc1 and other coronaviruses.
(DOCX)

Table S4 Amino acid sequence identities of partial RdRp among type II CCoV and types I and II FCoV.
(DOCX)

Table S5 Amino acid sequence identities of partial S protein among type II CCoV and types I and II FCoV.
(DOCX)

Table S6 Amino acid sequence identities of N protein among type II CCoV and types I and II FCoV.
(DOCX)
