# Supplementary material for: Emergence of Pathogenic Coronaviruses in Cats by Homologous Recombination between Feline and Canine Coronaviruses
Source: PLoS One. 2014 Sep 2;9(9):e106534. doi: 10.1371/journal.pone.0106534 (PMC4152292; doi:10.1371/journal.pone.0106534)
Supplement: Table S1 — Primers used in this study. (DOCX) [file pone.0106534.s001.docx]

| Table S1. Primers used in this study | | | |
| --- | --- | --- | --- |
| Primer | Gene | Sequence (5' to 3') | Position^a^ |
| 1bF | ORF1b | TTGATTCAAAGATTTGAGTATTGG | 1-24 |
| 1bR | S | AGGCACAATGTAAGCACAATCATGG | 379-375 |
| 1bFF1 | ORF1b | AACGTGCCATGATTGTGC | 349-366 |
| 1bF2F | S | GTGGTTATTACCCTACAGAG | 526-545 |
| 1bF2R | S | CTCTGTAGGGTAATAACCAC | 526-545 |
| 1bFF2 | S | GCTCAAGTACTGCCACAT | 1006-1023 |
| 1bFR2 | S | CATATGCAGCACTGTGTG | 1044-1027 |
| CCVSF | S | AGCACTTTTCCTATTGATTG | 1686-1705 |
| CCVSR | S | GTTAGTTTGTCTAATAATACCAACACC | 2483-2457 |
| CCVScenF | S | TAAGTAACATCACACTACC | 2026-2044 |
| CCVScenR | S | CCAGTTTTTATAACAGCTG | 2194-2176 |
| CCVScenRR2 | S | AACAGTAACGCGGTCCAT | 1572-1555 |
| CCVScenRF2 | S | TACAGTGAGCGAGTCAAG | 1499-1516 |
| CCVScenRF3 | S | GCATACATTAGTGGCCGT | 930-947 |
| CCVScenRR3 | S | CAGTACTTGAGCGAGAGT | 1017-1000 |
| CCVScenRF4 | S | GCTCATACCACATTGCTTCG | 391-410 |
| CCVScenRR4 | S | CGACGTACTCGAAGCAAT | 419-402 |
| CCVScenRF5 | S | AACATGGCACAAGAGTGCTG | 1019-1038 |
| CCVScenRR5 | S | CAGGTTGTAAGACTGACCAC | 959-940 |
| SF2 | S | TCTTGGTATGAAGCGTAGTGG | 1979-1999 |
| SR2 | S | TACCAATAGCTTGATTGAAAGC | 3591-3570 |
| SR3 | S | GCAGTTAGGTGGCTTAAAGC | 3727-3708 |
| S2cenF | S | CTATTCTGTGACACCATGTG | 2564-2583 |
| S2cenR | S | GCGCTTGCTCAATAGTTTGA | 3039-3020 |
| S2cenFF1 | S | CATCTGTTGAGGCGTTCA | 3115-3132 |
| S2cenFR1 | S | CTAGCCAAGAACCACCTA | 3195-3178 |
| S2cenFF2 | S | GAGTGCTGATGCACAAGT | 3797-3814 |
| S2cenFR2 | S | TCAGCCTGTCAACTTGTG | 3825-3808 |
| S2cenFF3 | S | CTGGACTGTACCTGAATTG | 4343-4361 |
| S2cenFR3 | S | GTGTCAATTCAGGTACAG | 4365-4348 |
| S2cenFF4 | 3a | GACACACTTCTTGAGGCT | 4919-4936 |
| S2cenFR4 | 3a | TGGAGAGACCAAGCTTAG | 4970-4953 |
| S2cenFR5 | S | AGTCTACAACACGTCTTCTAC | 4689-4669 |
| NF | M | CTAAAGCTGGTGATTACTCAACAG | 6847-6870 |
| NR | 7a | TAATAAATACAGCGTGGAGGAAAAC | 8119-8095 |
| NcenF | N | AGAGGAAGGCAACAATCCAA | 7464-7483 |
| NcenR | N | CCTGCAGTTCTCTTCCAGGT | 7660-7641 |
| N-R | N | CACCATCCTTTGCAACCCAG | 7293-7274 |
| N-R-2 | N | CAGATCTAGGCTGAGAACCA | 7440-7421 |
| N1 | N | MMAAYAAACACACCTGGAAG | 7630-7649 |
| N4 | N | CATCTCAACCTGTGTGTCAT | 8063-8044 |
| N-RF1 | M | TGGCCTTACCATCGATCA | 6719-6736 |
| N-RR1 | M | CGATGGTTCTACTAGGTG | 6780-6763 |
| N-RR2 | M | GCGCAATAACGTTCACCA | 6196-6179 |
| N-RR2-2 | E | GCTTCGTCGGGATTATATGC | 6111-6092 |
| N-RF2 | M | AGCGTGTGCAATTGCATG | 6155-6172 |
| N-RF2-2 | E | CTTCTTCTGGCTCCTGTTGA | 5932-5951 |
| N-RR3 | Between 3c and E | GCCACCATACAATGTGAC | 5637-5620 |
| N-RF3 | Between 3c and E | GAGAAGTTCTCACAGCTC  36 | 5595-5612 |
| N-RF4 | 3c | AGTTCAGCATTGCTGTGCTC | 5319-5338 |
| N-FF | 7a | CTGACAGTAGTCTGCGTGTA | 8222-8241 |
| N-F2F | 7b | GCATCTAGAGTGTGCTCACA | 8881-8900 |
| NR-R | 7b | ACTCTCACACTCAACACGAG | 8605-8586 |
| 3-R | 3’ UTR | GTGTATCACTATCAAAAGGAATA | 9315-9293 |
| 52F | TRS | ACTAGCCTTGTGCTAGATTT |  |
| a: Position is shown based on the fc1 sequence. | | | |
