# Supplementary material for: Emergence of Pathogenic Coronaviruses in Cats by Homologous Recombination between Feline and Canine Coronaviruses
Source: PLoS One. 2014 Sep 2;9(9):e106534. doi: 10.1371/journal.pone.0106534 (PMC4152292; doi:10.1371/journal.pone.0106534)
Supplement: Table S2 — Comparison of ORF identities between C3663 and other coronaviruses. (DOCX) [file pone.0106534.s002.docx]

| Table S.2 Comparison of ORF identities between C3663 and other coronaviruses | | | | | | | | | | |
| --- | --- | --- | --- | --- | --- | --- | --- | --- | --- | --- |
|  | Identity with type I FCoV C3663 (%) (amino acids) | | | | | | | | | |
|  | RdRp | S | 3a | 3b | 3c | E | M | N | 7a | 7b |
| fc1 | **95.4** | 48.4 | 69.0 | 59.7 | 71.5 | 76.8 | 83.4 | 74.9 | 81.2 | 65.9 |
| M91-267 | **94.7** | 48.7 | 69.0 | 56.7 | 63.6 | 76.8 | **90.0** | **92.8** | **95.1** | **91.3** |
| KUK-H/L | **98.5** | 48.6 | 69.0 | 59.7 | 79.1 | 78.0 | **89.6** | **89.9** | **96.0** | **92.2** |
| Tokyo/cat/130627 | **98.5** | 49.2 | **90.0** |  | **91.1** | **93.9** | **92.4** | **91.8** | **95.1** | **87.9** |
| Bold numbers indicate that the identity is over 85% | | | | | | | | | | |
