# Supplementary material for: Emergence of Pathogenic Coronaviruses in Cats by Homologous Recombination between Feline and Canine Coronaviruses
Source: PLoS One. 2014 Sep 2;9(9):e106534. doi: 10.1371/journal.pone.0106534 (PMC4152292; doi:10.1371/journal.pone.0106534)
Supplement: Table S3 — Comparison of ORF identities between fc1 and other coronaviruses. (DOCX) [file pone.0106534.s003.docx]

| Table S3. Comparison of ORF identities between fc1 and other coronaviruses | | | | | | | | | | |
| --- | --- | --- | --- | --- | --- | --- | --- | --- | --- | --- |
|  | Identity with type II CCoV fc1 (%) (amino acids) | | | | | | | | | |
|  | RdRp | S | 3a | 3b | 3c | E | M | N | 7a | 7b |
| C3663 | **95.4** | 48.4 | 69.0 | 59.7 | 71.5 | 76.8 | 83.4 | 74.9 | 81.2 | 65.9 |
| M91-267 | **99.2** | **97.5** | **100** | **98.6** | **97.7** | **100** | **90.1** | 76.4 | 81.2 | 58.8 |
| KUK-H/L | **94.7** | **96.5** | **95.8** | **98.6** | **96.2** | **97.6** | **89.7** | 74.6 | 82.2 | 59.7 |
| Tokyo/cat/130627 | **94.7** | **94.6** | 68.6 |  | 74.6 | 78.0 | 83.5 | 76.4 | 82.2 | 59.3 |
| Bold numbers indicate that the identity is over 85% | | | | | | | | | | |
