# Supplementary material for: Emergence of Pathogenic Coronaviruses in Cats by Homologous Recombination between Feline and Canine Coronaviruses
Source: PLoS One. 2014 Sep 2;9(9):e106534. doi: 10.1371/journal.pone.0106534 (PMC4152292; doi:10.1371/journal.pone.0106534)
Supplement: Table S4 — Amino acid sequence identities of partial RdRp among type II CCoV and types I and II FCoV. (DOCX) [file pone.0106534.s004.docx]

| Table S4. Amino acid sequence identities of partial RdRp among type II CCoV and types I and II FCoV | | | | | |
| --- | --- | --- | --- | --- | --- |
|  | fc1 | C3663 | M91-267 | KUK-H/L | Tokyo/cat/130627 |
| fc4 | **100.0%** | **95.4%** | **99.2%** | 94.7% | 94.7% |
| fc7 | **100.0%** | **95.4%** | **99.2%** | 94.7% | 94.7% |
| fc9 | **100.0%** | **95.4%** | **99.2%** | 94.7% | 94.7% |
| fc76 | **100.0%** | **95.4%** | **99.2%** | 94.7% | 94.7% |
| fc100 | **100.0%** | **95.4%** | **99.2%** | 94.7% | 94.7% |
| fc97-022 | **99.2%** | **96.2%** | **98.5%** | **95.4%** | **95.4%** |
| fc94-039 | **100.0%** | **95.4%** | **99.2%** | 94.7% | 94.7% |
| fc00-016 | **100.0%** | **95.4%** | **99.2%** | 94.7% | 94.7% |
| fc00-089 | **100.0%** | **95.4%** | **99.2%** | 94.7% | 94.7% |
| Bold numbers indicate that the identity is over 95%. | | | | | |
