# Supplementary material for: Emergence of Pathogenic Coronaviruses in Cats by Homologous Recombination between Feline and Canine Coronaviruses
Source: PLoS One. 2014 Sep 2;9(9):e106534. doi: 10.1371/journal.pone.0106534 (PMC4152292; doi:10.1371/journal.pone.0106534)
Supplement: Table S5 — Amino acid sequence identities of partial S protein among type II CCoV and types I and II FCoV. (DOCX) [file pone.0106534.s005.docx]

| Table S5. Amino acid sequence identities of partial S protein among type II CCoV and types I and II FCoV | | | | | |
| --- | --- | --- | --- | --- | --- |
|  | fc1 | C3663 | M91-267 | KUK-H/L | Tokyo/cat/130627 |
| fc4 | **96.1%** | 29.3% | **97.0%** | **96.5%** | **94.8%** |
| fc7 | **95.7%** | 29.3% | **96.5%** | **96.1%** | **94.3%** |
| fc9 | **93.9%** | 30.1% | **95.2%** | **94.3%** | **93.0%** |
| fc76 | **98.3%** | 27.8% | **96.1%** | **98.7%** | **95.7%** |
| fc100 | **95.2%** | 29.3% | **96.1%** | **95.7%** | **93.9%** |
| fc97-022 | **97.4%** | 29.3% | **95.2%** | **97.8%** | **97.0%** |
| fc94-039 | **98.3%** | 27.8% | **97.0%** | **98.7%** | **95.7%** |
| fc00-016 | **97.4%** | 28.8% | **95.2%** | **97.8%** | **96.5%** |
| fc00-089 | **97.8%** | 28.9% | **95.7%** | **98.2%** | **97.4%** |
| Bold numbers indicate that the identity is over 90%. | | | | | |
