# Supplementary material for: Emergence of Pathogenic Coronaviruses in Cats by Homologous Recombination between Feline and Canine Coronaviruses
Source: PLoS One. 2014 Sep 2;9(9):e106534. doi: 10.1371/journal.pone.0106534 (PMC4152292; doi:10.1371/journal.pone.0106534)
Supplement: Table S6 — Amino acid sequence identities of N protein among type II CCoV and types I and II FCoV. (DOCX) [file pone.0106534.s006.docx]

| Table S6. Amino acid sequence identities of N protein among type II CCoV and types I and II FCoV | | | | | |
| --- | --- | --- | --- | --- | --- |
|  | fc1 | C3663 | M91-267 | KUK-H/L | Tokyo/cat/130627 |
| fc4 | **98.2%** | 75.9% | 77.5% | 75.4% | 77.2% |
| fc7 | **98.2%** | 75.9% | 77.5% | 75.4% | 77.2% |
| fc9 | **97.9%** | 75.4% | 77.0% | 75.4% | 77.0% |
| fc76 | **97.9%** | 75.9% | 77.5% | 75.7% | 77.5% |
| fc100 | **97.6%** | 75.7% | 77.2% | 75.4% | 77.2% |
| fc97-022 | **96.9%** | 75.4% | 76.7% | 74.6% | 76.4% |
| fc94-039 | **97.6%** | 74.6% | 75.9% | 74.6% | 76.2% |
| fc00-016 | **96.9%** | 75.1% | 76.4% | 74.6% | 76.4% |
| fc00-089 | **96.6%** | 74.6% | 75.9% | 74.1% | 75.9% |
| Bold numbers indicate that the identity is over 90%. | | | | | |
